# Supplementary figures and images for: Peritoneal dialysis modality transition and impact on phosphate and potassium serum levels
Source: PLoS One. 2021 Oct 15;16(10):e0257140. doi: 10.1371/journal.pone.0257140 (PMC8519456; doi:10.1371/journal.pone.0257140)

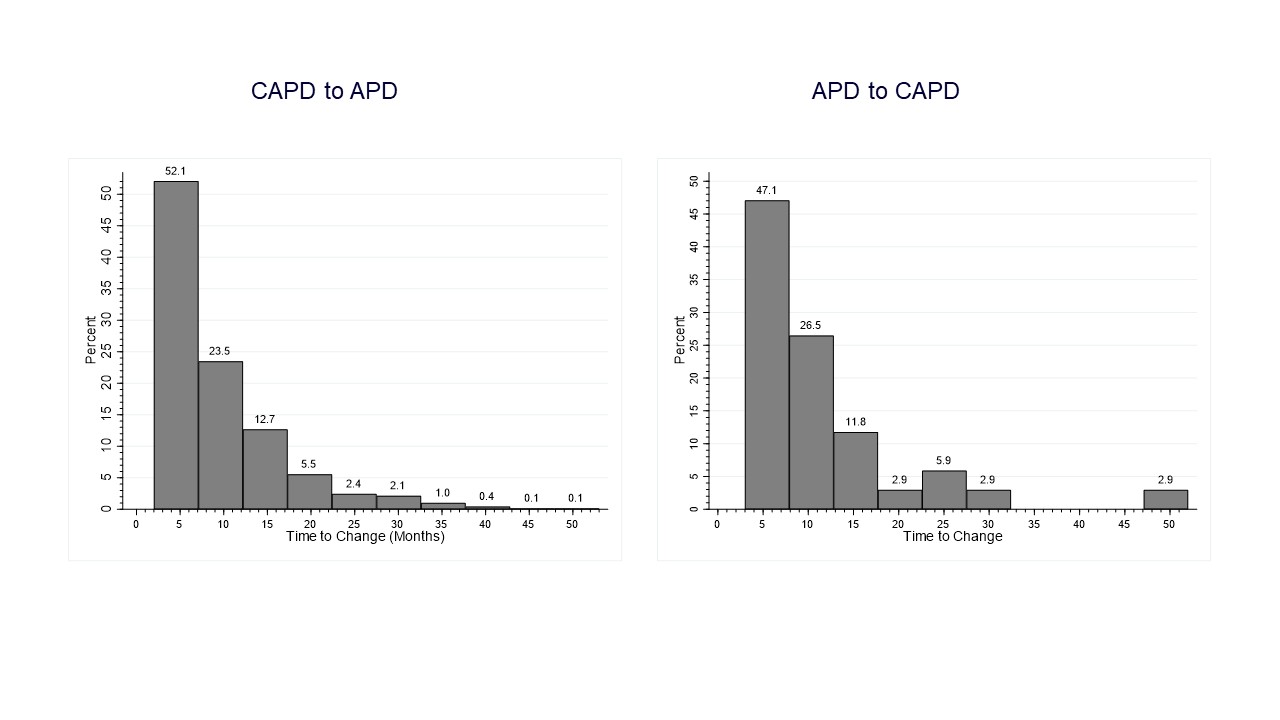

Supplement: S1 Fig — (JPG) [file pone.0257140.s001.jpg]

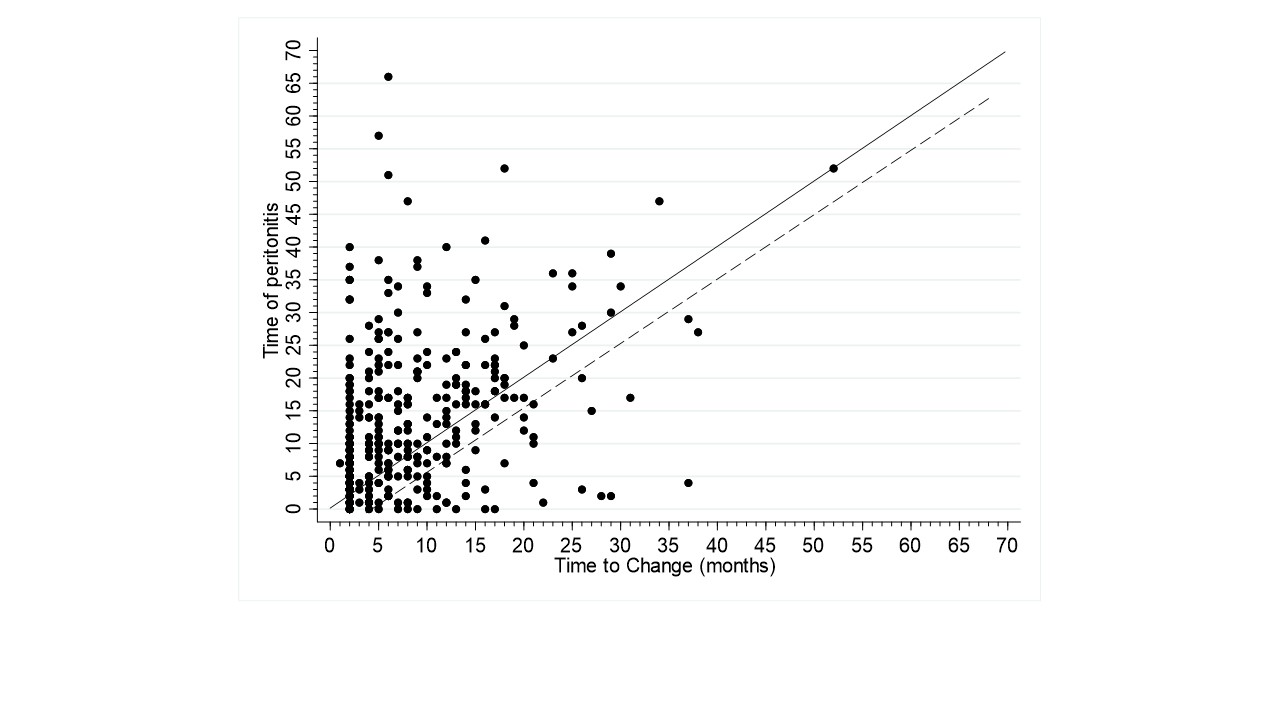

Supplement: S2 Fig — All dots above the continuous black line represents peritonitis episodes that occurred after modality change. All dots below the dashed black line are peritonitis episodes that occurred at least 3 months before modality change. (JPG) [file pone.0257140.s002.jpg]
