# Supplementary material for: Peritoneal dialysis modality transition and impact on phosphate and potassium serum levels
Source: PLoS One. 2021 Oct 15;16(10):e0257140. doi: 10.1371/journal.pone.0257140 (PMC8519456; doi:10.1371/journal.pone.0257140)
Supplement: Attachment — Submitted filename: 1-s2.0-S0085253815495948-main.pdf [file pone.0257140.s003.pdf]

Kidney International, Vol. 64 (2003), pp. 2238–2243

Removal of middle molecules and protein-bound solutes by peritoneal dialysis and relation with uremic symptoms

Bert Bammens, Pieter Evenepoel, Kristin Verbeke, and Yves Vanrenterghem

Department of Medicine, Division of Nephrology and Laboratory of Radiopharmaceutical Chemistry, University Hospital Gasthuisberg, Leuven, Belgium

Removal of middle molecules and protein-bound solutes by peritoneal dialysis and relation with uremic symptoms.

Background. Current guidelines for peritoneal dialysis adequacy are based on kinetics of small water-soluble molecules and do not consider the role of other compounds such as middle molecules and protein-bound solutes. Information on the elimination characteristics of the latter solutes by peritoneal dialysis is limited. Moreover, their relation with uremic symptoms remains unclear. The aim of the present study was (*1*) to investigate the relative contribution of residual renal function to the overall clearances of β_2-microglobulin (β_2m), a middle molecule, and p-cresol, a protein-bound solute, in adults on peritoneal dialysis as compared to small water-soluble molecules and (*2*) to evaluate relations between serum levels and uremic symptoms.

Methods. We performed a cross-sectional observational study, including 30 nonanuric peritoneal dialysis patients. Total, peritoneal, and renal clearances were calculated for urea nitrogen (60 D), creatinine (113 D), phosphate (96 D), β_2m (11.8 kD), and p-cresol (108 D). All patients were asked to complete a uremic symptom questionnaire.

Results. Declining total clearances (L/week/1.73 m^2) were measured for urea nitrogen, creatinine, phosphate, β_2m, and p-cresol, respectively: 97.3 ± 4.6, 98.9 ± 6.1, 64.0 ± 3.4, 23.1 ± 2.6, and 17.5 ± 2.3 (Friedman test $P < 0.001$). Conversely, the contribution of residual renal function (%) to the respective solute clearances increased significantly: 31.6 ± 3.2, 51.0 ± 4.0, 42.4 ± 4.0, 68.0 ± 5.4, 61.9 ± 4.6 (Friedman test $P < 0.001$). The serum level of p-cresol, but of none of the other solutes examined, correlated significantly with the symptom score (Pearson $r = 0.48$, $P = 0.008$).

Conclusion. During peritoneal dialysis p-cresol behaves like β_2m, probably due to its protein binding. The total clearance of both molecules is significantly lower as compared to water-soluble solutes and mainly depends on residual renal function. Our data further suggest that protein-bound solutes are involved in the pathophysiology of uremic symptoms.

Key words: peritoneal dialysis, protein-bound solutes, residual renal function, symptoms.

Received for publication April 11, 2003
and in revised form June 20, 2003
Accepted for publication July 17, 2003

© 2003 by the International Society of Nephrology

Defining the optimal dose of dialysis for end-stage renal disease (ESRD) patients treated with peritoneal dialysis has been among the principle goals of the nephrology community for more than two decades. The currently used guidelines suggest that, for continuous ambulatory peritoneal dialysis (CAPD), the delivered dose should be a total Kt/V for urea nitrogen of at least 2.0 per week and a total clearance of creatinine of at least 60 L/week/1.73 m^2 for high and high-average transporters, and 50 L/week/1.73 m^2 for low and low-average transporters [1, 2]. The guidelines were based mainly on the results of the landmark CANUSA Study [3] and assume that peritoneal and renal clearances contribute equally to patient outcome. Subsequent analysis of the CANUSA data [4], the recent ADEMEX trial [5] and other studies [6, 7], however, have cast doubt on this assumption and have emphasized the importance of residual renal function as a predictor of survival. In this regard, several investigators have stressed the role of renal function in the maintenance of an adequate fluid balance [8–11]. However, the superiority of renal over peritoneal clearance for predicting patient outcome might also be explained by the finding that the elimination of some uremic retention solutes depends largely on renal metabolism and/or secretion at the tubular level, which cannot be equaled by increasing peritoneal transport. This is the case for the middle molecule β_2-microglobulin (β_2m) as well as for the recently recognized group of protein-bound solutes [12]. β_2m is filtered in the glomeruli and reabsorbed and degraded in the proximal tubular cells. Its renal catabolism in a healthy subject was found to be 150 to 220 mg/day [13]. Protein-bound solutes are mainly excreted by tubular secretion via the organic anion transport system of the proximal tubular cells [14]. p-Cresol, a fermentation metabolite of the amino acid tyrosine, is regarded as a prototype of the protein-bound solutes [15]. It has been demonstrated in vitro to inhibit several biochemical, biologic, and physiologic functions [15–17]. However, except for one recent report in hemodialysis patients [18], no correlations with clinical end points have been presented to date.

The aim of this cross-sectional observational study was (*1*) to investigate the relative contribution of residual renal function to the overall clearances of β_2m and p-cresol in adults on peritoneal dialysis as compared to small water-soluble solutes and (*2*) to evaluate the relation between the serum level of individual retention solutes and the presence of well-recognized uremic symptoms.

METHODS

Patients and study design

We performed a single-center cross-sectional observational study. Thirty nonanuric patients with ESRD treated with peritoneal dialysis at the University Hospital Leuven were included. Fifteen patients were on automated peritoneal dialysis (APD) and 15 were on CAPD. The causes of ESRD were diabetic nephropathy ($N = 5$), polycystic kidney disease ($N = 4$), glomerular disease ($N = 12$), tubulointerstitial disease ($N = 4$), and unknown etiologies ($N = 5$). Demographic (age, gender, weight, and length) and clinical data (dialysis duration, medication, and comorbidity) were collected by reviewing the medical records. Comorbidity was scored according to Davies et al [19] and reported as low, medium or high grade. For the determination of clearances of urea nitrogen, creatinine, phosphate, p-cresol,and β_2m a mid-day blood sample was taken and total amounts of urine and peritoneal drainage were collected during the preceding 24-hour period, weighed, and sampled. All samples were stored at −80°C until analysis. For the evaluation of the relation between the serum level of individual solutes and uremic symptoms, the patients were asked to complete a uremic symptom questionnaire. The study was approved by the Ethical Committee of the University Hospital Leuven and informed consent was obtained from all patients.

Analytic methods

Urea nitrogen, creatinine, and phosphate were measured by standard laboratory techniques. β_2m was quantitated by rate nephelometry using an Immage Instrument (Beckman Coulter, Brea, CA, USA). p-Cresol was analyzed by gas chromatography mass spectrometry (GC-MS) technology. Five hundred µL of serum was diluted with 450 µL water. The pH of a 950 µL sample (diluted serum, urine or dialysis fluid) was adjusted to pH 1 with concentrated H_2SO_4 and the solution was heated to 90°C for 30 minutes. After a cooling down period to ambient temperature, 50 µL 2,6-dimethylphenol solution (20 mg/100 mL) was added as internal standard. One milliliter ethyl acetate was added for the extraction of p-cresol. The solution was well mixed during 30 seconds and centrifuged at 3300 rpm for 20 minutes. Then, 500 µL of the supernatant was dried over anhydrous sodium sulfate and 100 µL of the resultant sample was transferred to the GC-MS (Trace GC-MS, Thermofinnigan, San José, CA, USA) for automatic splitless injection of 0.5 µL. The analytic column used was a 30 m × 0.32 mm internal diameter, film thickness 1 µm AT5-MS (Alltech, Deerfield, IL, USA). Helium GC grade was used as a carrier gas with a constant flow of 1.3 mL/min. The oven was programmed from 75°C (isotherm for 5 minutes) to 280°C with 15°C per minute. After separation, p-cresol was identified by MS (Electron Impact full scan mode from m/z 59 to m/z 590 at 2 scan/second). Quantitative results were obtained by the internal standard method and calculated as concentrations (mg/L).

Calculations

Peritoneal, renal, and total clearances normalized to 1.73 m^2 body surface area (BSA) (L/week/1.73 m^2) were calculated for all solutes by direct determination from dialysis fluids, urine and mid-day serum solute concentrations. According to the National Kidney Foundation Dialysis Outcomes Quality Initiative (NKF-K-DOQI) guidelines [2], residual glomerular filtration rate (GFR) was estimated by calculating the arithmetic mean of renal urea nitrogen and creatinine clearance and expressed in mL/min/1.73 m^2. Peritoneal, renal, and total Kt/V were calculated only for urea nitrogen (Kt/V_{UN}). BSA was estimated by the Du Bois and Du Bois method [20]. The distribution volume of urea nitrogen (V) was assessed by the Watson, Watson, and Batt formula for total body water [21]. Protein nitrogen appearance, normalized to body weight (nPNA) was calculated according to Bergström, Heimburger, and Lindholm [22].

Uremic symptom score

A uremic symptom score was developed, using a previously described dyspepsia questionnaire [23–25] with the addition of 12 symptoms. Each patient was asked to grade the intensity (0, absent; 1, mild; 2, relevant; 3, severe and interfering with daily activities) of 20 different symptoms (Table 1). As indicated in the table, the total sum of scores (S_{tot}) was calculated as well as the subtotals for three different symptom classes: gastrointestinal symptoms (S_{gi}), neurological symptoms (S_{neur}), and itching (S_{skin}).

Statistics

Data are expressed as mean ± SEM. Differences between APD and CAPD were evaluated using unpaired Student *t* test or Mann-Whitney *U* test for continuous data and chi-square test of association for categorical data where appropriate. For paired comparisons of different solute clearances, Friedman test with post hoc analysis was used. Pearson correlation coefficients were calculated to assess possible associations between clearances, parameters of renal function, serum solute concentrations, and uremic symptom scores. *P* values less than 0.05

Table 1. Uremic symptom questionnaire

	0 1 2 3		
1. Epigastric discomfort worsening with food intake	0 0 0 0		
2. Epigastric pain	0 0 0 0		
3. Early satiety	0 0 0 0		
4. Postprandial fullness	0 0 0 0		
5. Bloating	0 0 0 0		
6. Nausea	0 0 0 0	S_{gi} (0–33)	
7. Vomiting	0 0 0 0		
8. Belching	0 0 0 0		
9. Epigastric burning	0 0 0 0		
10. Hiccup	0 0 0 0		
11. Decreased appetite	0 0 0 0		S_{tot} (0–60)
12. Sleeping disorders	0 0 0 0		
13. Fatigue	0 0 0 0		
14. Erratic memory	0 0 0 0		
15. Concentration disturbances	0 0 0 0		
16. Drowsiness	0 0 0 0	S_{neur} (0–24)	
17. Headache	0 0 0 0		
18. Cramps	0 0 0 0		
19. Restless legs	0 0 0 0		
20. Itching	0 0 0 0	S_{skin} (0–3)	

Indicate the intensity of each of the following symptoms during the previous 3 months 0 = absent 1 = mild 2 = relevant 3 = severe and interfering with daily activities.

Example of the uremic symptom questionnaire. In the table, total sum of scores and the subtotals according to three symptom classes are indicated. The ranges of possible scores are indicated between parentheses.

were considered significant. The SAS version 8.02 (SAS Institute, Cary, NC, USA) software program was used for the statistical analysis.

RESULTS

Patient characteristics

Patient characteristics are summarized in Table 2. Mean age was 52.0 ± 3.0 years. Mean duration of dialysis at the time of the study was 16.2 ± 2.0 months. Mean body weight was 66.5 ± 2.2 kg and mean BSA was 1.75 ± 0.04 m^2. Mean 24-hour urine output was 1009 ± 120 mL. Mean 24-hour peritoneal drainage was 11658 ± 553 mL. There were no statistically significant differences between APD and CAPD patients in any of the observed parameters. Renal diagnoses were equally distributed between the two groups. There were no differences in the use of diuretics, angiotensin-converting enzyme (ACE) inhibitors, angiotensin II receptor blockers, other antihypertensives, and nonsteroidal anti-inflammatory drugs.

Peritoneal dialysis adequacy

No significant differences were found between APD and CAPD for mid-day serum levels of urea nitrogen, creatinine, phosphate, and β_2m. Serum p-cresol was significantly lower in CAPD than in APD patients (P = 0.0049). Peritoneal, renal, total Kt/V_{UN} values, and GFR were not different between the two groups (Table 3).

There were no differences between APD and CAPD in peritoneal clearances of urea nitrogen, creatinine, and phosphate. Conversely, peritoneal clearances of β_2m (2.83 ± 0.21 vs. 6.24 ± 0.83, P = 0.003) and p-cresol (3.60 ± 0.37 vs. 5.91 ± 0.69, P = 0.020) were significantly lower in APD than in CAPD patients. No differences in renal clearances between APD and CAPD were observed for any of the solutes studied (data not shown).

Total and separated (peritoneal and renal) clearance values for the 30 patients are summarized in Table 4 and illustrated in Figure 1. Data are also expressed as percentages of the overall clearances. Declining total clearances were measured for urea nitrogen, creatinine, phosphate, β_2m, and p-cresol, respectively (Friedman test $P < 0.001$). Conversely, the contribution of residual renal function to the respective solute clearances increased significantly (Friedman test $P < 0.001$).

Serum levels of β_2m and p-cresol correlated negatively with 24-hour urine volume ($r = -0.46$, $r = -0.15$, respectively), renal weekly Kt/V_{UN} ($r = -0.42$, $r = -0.27$, respectively) and GFR ($r = -0.49$, $r = -0.32$, respectively). However, statistical significance was reached only for β_2m. Peritoneal clearance values of urea nitrogen, creatinine, and phosphate were inversely correlated with their respective renal clearances ($r = -0.43$, $P = 0.019$; $r = -0.32$, $P = 0.082$; and $r = -0.43$, $P = 0.024$, respectively). This relationship was absent for β_2m ($r = -0.19$, $P = 0.342$) and p-cresol ($r = 0.09$, $P = 0.625$).

Uremic symptom score

For the total group of 30 patients mean S_{tot} was 7.6 ± 1.2. S_{gi}, S_{neur}, and S_{skin} amounted to 2.63 ± 0.78, 4.27 ± 0.50, and 0.73 ± 0.16, respectively. There were no significant differences between APD and CAPD for any of the score sums, although there was a trend to lower scores in CAPD patients.

Of all solutes, only the serum level of p-cresol correlated significantly with S_{gi} ($r = 0.47$, $P = 0.008$), S_{skin} ($r = 0.38$, $P = 0.041$), and S_{tot} ($r = 0.48$, $P = 0.008$) (Fig. 2). There were no significant differences in symptom scores between patients with low, medium, and high grade comorbidity.

DISCUSSION

Our study, comprising 30 ESRD patients treated with peritoneal dialysis, shows that the total and peritoneal clearances of β_2m, p-cresol, and phosphate are significantly lower as compared to the clearances of the reference uremic retention solutes urea nitrogen and creatinine (Table 4) (Fig. 1). For β_2m this observation is not surprising, since the high molecular weight of the solute (11815 D) hampers its diffusive and convective transport through the pores of the peritoneal membrane [26–28]. The findings are in accordance with earlier studies in children [29, 30] and adults on peritoneal dialysis [31]. The low clearances of p-cresol (108 D) represent a new finding. They are not unexpected, however, given the

Table 2. Patient characteristics

	All	APD	CAPD	*P* value
Number	30	15	15	—
Age *years*	52.0 ± 3.0	52.5 ± 5.1	51.5 ± 3.5	0.805
Male/female	16/14	8/7	8/7	1.000
Dialysis duration *months*	16.2 ± 2.0	19.7 ± 3.3	12.6 ± 2.0	0.070
24-hour urine output *mL*	1009 ± 120	1096 ± 197	922 ± 140	0.525
24-hour peritoneal drainage *mL*	11658 ± 553	12607 ± 832	10709 ± 669	0.092
24-hour ultrafiltration volume *mL*	1308 ± 339	874 ± 355	1742 ± 569	0.206
Body weight *kg*	66.5 ± 2.2	65.6 ± 3.5	66.5 ± 2.8	0.566
Body surface area m^2	1.75 ± 0.04	1.74 ± 0.06	1.76 ± 0.05	0.789
Normalized protein nitrogen appearance *g/kg/day*	2.06 ± 0.10	2.01 ± 0.12	2.11 ± 0.16	0.886
Comorbidity (low/medium/high grade)	15/11/4	8/4/3	7/7/1	0.390
Use of phosphate binders (yes/no)	16/4	13/2	13/2	1.000
Use of vitamin D (yes/no)	11/19	4/11	7/8	0.256

Abbreviations are: APD, automated peritoneal dialysis; CAPD, continuous ambulatory peritoneal dialysis.
Date are expressed as mean ± SEM. Differences between APD and CAPD were evaluated using unpaired Student *t* test or Mann-Whitney *U* test for continuous data and chi-square test of association for categoric data where appropriate.

Table 3. Mid-day serum concentrations, Kt/V_{UN}, GFR

	All	APD	CAPD	*P* value
Number	30	15	15	—
UN *mg/dL*	110.6 ± 4.7	111.2 ± 6.2	101.1 ± 7.3	0.712
Cr *mg/dL*	7.18 ± 0.39	7.27 ± 0.44	7.08 ± 0.66	0.499
P *mg/dL*	4.46 ± 0.21	4.72 ± 0.34	4.20 ± 0.24	0.318
p-cresol *mg/L*	37.2 ± 3.2	46.5 ± 4.8	27.9 ± 2.5	**0.005**
β_2m *mg/L*	18.1 ± 1.4	17.8 ± 1.5	18.4 ± 2.4	0.637
Peritoneal Kt/V_{UN}	1.94 ± 0.16	1.76 ± 0.16	2.13 ± 0.27	0.281
Renal Kt/V_{UN}	0.88 ± 0.10	0.87 ± 0.14	0.89 ± 0.15	0.853
Total KT/V_{UN}	2.83 ± 0.16	2.63 ± 0.11	3.02 ± 0.30	0.453
GFR *mL/min/1.73* m^2	4.17 ± 0.47	4.03 ± 0.63	4.32 ± 0.72	0.789

Abbreviations are: APD, automated peritoneal dialysis; CAPD, continuous ambulatory peritoneal dialysis; GFR, glomerular filtration rate; UN, urea nitrogen; Cr, creatinine; P, phosphate; β_2m, β_2-microglobulin.
Data are expressed as mean ± SEM. Differences between APD and CAPD were evaluated using Mann-Whitney *U* test. Total p-cresol was significantly lower in CAPD than in APD.

molecules' protein binding, which was demonstrated to be virtually complete in healthy controls and about 90% in ESRD patients [32]. The protein binding also explains the limited removal of the solute by low- and high-flux hemodialysis, as demonstrated by others [32, 33].

Our clearance data demonstrate that the elimination of β_2m and p-cresol depends largely on residual renal function (Table 4) (Fig. 1). The importance of renal elimination is further stressed by the inverse relationship found between serum levels of both molecules and parameters of renal function (24-hour urine volume, renal Kt/V_{UN}, and GFR). For p-cresol this relationship lacks statistical significance, which can probably be attributed to the fact that the generation rate of the molecule, in contrast to that of β_2m [34–36], can vary substantially within and between individuals. The amount of ingested protein escaping small intestinal digestion and absorption, the characteristics of the bacterial flora and the colonic transit time were all shown to influence p-cresol generation [37–39]. Variations of these factors might obscure the relationship between serum levels of the solute and its renal elimination.

Peritoneal clearance values of urea nitrogen, creatinine, and phosphate were inversely correlated with their respective renal clearances, which was not the case for β_2m and p-cresol. This observation further underlines the importance of residual renal function for the elimination of the latter molecules, since a loss of renal clearance seems not to be compensated by an increase in peritoneal clearance.

Several recent studies indicate the value of residual renal function as a predictor of patient survival in peritoneal dialysis patients [4–7]. This has been explained by its role in the maintenance of an adequate fluid balance [8–11]. In view of our findings, the conservation of renal elimination mechanisms other than glomerular filtration [13, 14] might contribute substantially to the explanation of residual renal function as an outcome predictor.

The finding of low clearance values of phosphate (96 D) is in accordance with earlier reports illustrating the middle molecule characteristics of the solute [40–42]. Several explanations for this particular behavior have been formulated. First, due to its hydrophilic characteristic, the phosphate molecule is surrounded by an aqueous cover. Moreover, up to 40% of the circulating phosphate has been shown to be a component of sodium, calcium, and magnesium salts [40]. Finally, phosphate is mainly distributed in the intracellular space with a slow intra- and extracellular solute transfer rate [43].

We found significant differences in peritoneal clearances of β_2m and p-cresol between APD and CAPD. This observation corroborates the results of a recent study, which showed that in contrast to small water-soluble molecules, the peritoneal clearance of β_2m depends mainly on the total dwell hours of peritoneal dialysis and not on the number of exchanges of dialysis fluid [31]. Moreover, although not statistically significant, the 24-hour ultrafiltration volume was higher in CAPD than in APD patients, which might have contributed to a greater solute removal by convection in the former. On the other hand, the duration of dialysis at the time of our study was shorter in the CAPD patients (not statistically significant). This may have biased our results, since the

Table 4. Peritoneal, renal, and total clearances

	Urea nitrogen	Creatinine	Phosphate	β_2-microglobulin	p-cresol
Molecular weight *daltons*	60	113	96	11815	108
Total clearance *L/week/1.73 m*^{2}	97.3 ± 4.6	98.9 ± 6.1	$64.0 \pm 3.4^{a,b}$	$23.1 \pm 2.6^{a,b,c}$	$17.5 \pm 2.3^{a,b,c}$
Peritoneal clearance *L/week/1.73 m*^{2}	66.6 ± 4.8	45.3 ± 3.5^{a}	35.9 ± 2.7^{a}	$4.66 \pm 0.54^{a,b,c}$	$4.75 \pm 0.44^{a,b,c}$
Peritoneal clearance % of total clearance	68.4 ± 3.2	49.0 ± 4.0^{a}	57.6 ± 4.0^{a}	$32.0 \pm 5.4^{a,b,c}$	$38.1 \pm 4.6^{a,c}$
Renal clearance *L/week/1.73 m*^{2}	30.7 ± 3.5	53.6 ± 6.2^{a}	28.1 ± 3.6^{b}	$18.4 \pm 2.7^{a,b,c}$	$12.7 \pm 2.2^{a,b,c}$
Renal clearance % of total clearance	31.6 ± 3.2	51.0 ± 4.0^{a}	42.4 ± 4.0^{a}	$68.0 \pm 5.4^{a,b,c}$	$61.9 \pm 4.6^{a,c}$

Data are expressed as mean ± SEM. Differences were evaluated using Friedman test with post hoc analysis.
[a] $P < 0.05$ vs. urea nitrogen; [b] $P < 0.05$ vs. creatinine; [c] $P < 0.05$ vs. phosphate.

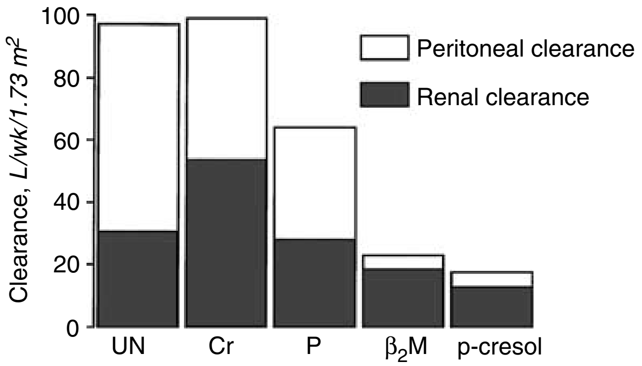

Fig. 1. Peritoneal, renal, and total clearances of urea nitrogen (UN), creatinine (Cr), phosphate (P), β_2-microglobulin (β_2m), and p-cresol. Mean values are illustrated.

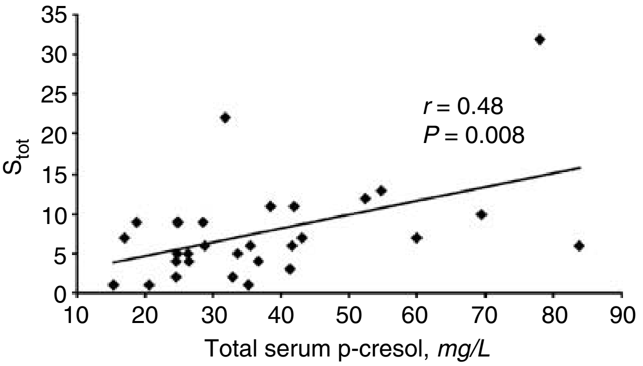

Fig. 2. Total sum of scores (S_{tot}) vs. total serum p-cresol (mg/L). Pearson correlation coefficient (r) and P value are indicated.

peritoneal membrane restriction coefficient for macromolecules has been demonstrated to increase in relation to the years on peritoneal dialysis treatment [30, 44].

Serum levels of p-cresol were significantly lower in CAPD than in APD patients. This finding most probably reflects the differences in peritoneal clearance of the molecule, since no differences between the two groups were seen in parameters of renal function (24-hour urine output, renal Kt/V_{UN}, or GFR) or in dietary protein intake (nPNA).

An interesting finding of our data is the positive correlation between serum levels of p-cresol and a uremic symptom score, based on a validated dyspepsia questionnaire [23–25]. To our knowledge, this is the first evidence of a clinical end point related to the protein-bound retention product in peritoneal dialysis patients. One might argue that this relation is self-evident, since p-cresol is just one of the numerous retention solutes in uremia and might play the role of "innocent bystander." However, p-cresol was the only of the five toxins studied revealing a significant correlation with the symptom score. Moreover, there was a trend to lower symptom scores in the CAPD patients in conjunction with lower serum levels and higher peritoneal clearances of p-cresol as compared to the APD patients. These observations further suggest that protein bound solutes play a role in the pathophysiology of uremic symptoms.

We recognize that the serum levels of p-cresol found by our laboratory are higher than most other values reported in the literature [45, 46]. A possible explanation for this discrepancy lies in the sample preparation, which consists essentially of a deproteinization and an extraction step. In addition to acid deproteinization, which is applied in most other studies, we used heat denaturation. It is not excluded that the high temperature used in this setting not only deproteinizes the serum but, in addition, provokes hydrolysis of circulating conjugates of p-cresol (p-cresylsulfate and p-cresylglucuronide). Although differences in concentration measurements would probably have no impact on clearance calculations, further research is needed to elucidate this topic.

CONCLUSION

Our data show clear evidence for the middle molecule characteristics of the protein-bound solute p-cresol during peritoneal dialysis. As for β_2m, its total and peritoneal clearances were shown to be lower than those of the small water-soluble solutes urea nitrogen and creatinine. Moreover, the importance of residual renal function in the elimination of β_2m and p-cresol was demonstrated. Finally, our study is supportive for the involvement of protein-bound solutes in the pathophysiology of uremic symptoms.

ACKNOWLEDGMENTS

We are grateful to X. Bossuyt, T. Coopmans, M. Dekens, V. De Preter, and A. Luypaerts for their excellent assistance. We also wish to thank all the participating patients and the nurses and physicians taking care of them. The study was supported by the FWO grant No. 1127602N.

Reprint requests to Pieter Evenepoel, M.D., Ph.D., Department of Medicine, Division of Nephrology, University Hospital Gasthuisberg, Herestraat 49, B-3000 Leuven, Belgium.
E-mail: bert.bammens@uz.kuleuven.ac.be

REFERENCES

1. National Kidney Foundation: NKF-DOQI Clinical Practice Guidelines for Peritoneal Dialysis Adequacy. *Am J Kidney Dis* 30(Suppl 2):S67–S136, 1997
2. National Kidney Foundation: NKF-K/DOQI Clinical Practice Guidelines for Peritoneal Dialysis Adequacy: Update 2000. *Am J Kidney Dis* 37(Suppl 1):S65–S136, 2001
3. CANUSA PD study group: Adequacy of dialysis and nutrition in continuous peritoneal dialysis: Association with clinical outcomes. *J Am Soc Nephrol* 7:198–207, 1996
4. Bargman JM, Thorpe KE, Churchill DN, for the CANUSA PD study group: Relative contribution of residual renal function and peritoneal clearance to adequacy of dialysis: A reanalysis of the CANUSA study. *J Am Soc Nephrol* 12:2158–2162, 2001
5. Paniagua R, Amato D, Vonesh E, *et al*: Effects of increased peritoneal clearances on mortality rates in peritoneal dialysis: ADEMEX, a prospective, randomized, controlled trial. *J Am Soc Nephrol* 13:1307–1320, 2002
6. Ates K, Nergizoglu G, Keven K, *et al*: Effect of fluid and sodium removal on mortality in peritoneal dialysis patients. *Kidney Int* 60:767–776, 2001
7. Chung SH, Heimbürger O, Stenvinkel P, *et al*: Association between residual renal function, inflammation and patient survival in new peritoneal dialysis patients. *Nephrol Dial Transplant* 18:590–597, 2003
8. Wang T, Lindholm B: Beyond CANUSA, DOQI, ADEMEX: What's next? *Perit Dial Int* 22:555–562, 2002
9. Heimbürger O: The negative results of the ADEMEX study may be positive for peritoneal dialysis. Time for a paradigm shift in the focus of peritoneal dialysis adequacy. *Perit Dial Int* 22:546–548, 2002
10. Piraino B: ADEMEX: How should it change our practice? *Perit Dial Int* 22:552–554, 2002
11. Churchill DN: The ADEMEX study: Make haste slowly. *J Am Soc Nephrol* 13:1415–1418, 2002
12. Vanholder R, Argiles A, Baurmeister U, *et al*: Uremic toxicity: Present state of the art. *Int J Art Organs* 24:695–725, 2002
13. Karlsson FA: Physical-chemical properties of β_2-microglobulin. *Immunochemistry* 11:111–117, 1974
14. Enomoto A, Takeda M, Tojo A, *et al*: Role of organic anion transporters in the tubular transport of indoxyl sulfate and the induction of its nephrotoxicity. *J Am Soc Nephrol* 13:1711–1720, 2002
15. Vanholder R, De Smet R, Lameire N: Protein bound uremic solutes: The forgotten toxins. *Kidney Int* 59(Suppl 78):S266–S270, 2001
16. Vanholder R, De Smet R, Waterloos MA, *et al*: Mechanisms of uremic inhibition of phagocyte reactive species production: Characterization of the role of p-cresol. *Kidney Int* 47:510–517, 1995
17. Dou L, Cerini C, Brunet P, *et al*: P-cresol, a uremic toxin, decreases endothelial cell response to inflammatory cytokines. *Kidney Int* 62:1999–2009, 2002
18. De Smet R, Van Kaer J, Van Vlem B, *et al*: Toxicity of free p-cresol: A prospective and cross-sectional analysis. *Clin Chem* 49:470–478, 2003
19. Davies SJ, Phillips L, Naish PF, Russell GI: Quantifying comorbidity in peritoneal dialysis patients and its relationship to other predictors of survival. *Nephrol Dial Transplant* 17:1085–1092, 2002
20. Du Bois D, Du Bois EF: A formula to estimate the approximate surface area if height and weight be known. *Arch Intern Med* 17:863–971, 1916
21. Watson PE, Watson ID, Batt RD: Total body water volumes for adult males and females estimated from simple anthropometric measurements. *Am J Clin Nutr* 33:27–39, 1980
22. Bergström J, Heimburger O, Lindholm B: Calculation of the protein equivalent of total nitrogen appearance from urea appearance. Which formulas should be used? *Perit Dial Int* 18:467–473, 1998
23. Tucci A, Corinaldesi R, Stanghellini V, *et al*: *Helicobacter pylori* infection and gastric function in patients with chronic idiopathic dyspepsia. *Gastroenterology* 103:768–774, 1992
24. Stanghellini V, Tosetti C, Paternico A, *et al*: Risk indicators of delayed gastric emptying of solids in patients with functional dyspepsia. *Gastroenterology* 110:1036–1042, 1996
25. Tack J, Piessevaux H, Coulie B, *et al*: Role of impaired gastric accommodation to a meal in functional dyspepsia. *Gastroenterology* 115:1346–1352, 1998
26. Flessner MF: Peritoneal transport physiology: Insights from basic research. *J Am Soc Nephrol* 2:122–135, 1991
27. Krediet RT, Koomen GCM, Koopman MG, *et al*: The peritoneal transport of serum proteins and neutral dextran in CAPD patients. *Kidney Int* 35:1064–1072, 1989
28. Imholz ALT, Koomen GCM, Struijk DG, *et al*: Effect of dialysate osmolarity on the transport of low-molecular weight solutes and proteins during CAPD. *Kidney Int* 43:1339–1346, 1993
29. Montini G, Amici G, Milan S, *et al*: Middle molecule and small protein removal in children on peritoneal dialysis. *Kidney Int* 61:1153–1159, 2002
30. Bouts AHM, Davin JC, Groothoff JW, *et al*: Standard peritoneal permeability analysis in children. *J Am Soc Nephrol* 11:943–950, 2000
31. Kim DJ, Ho Do J, Huh W, *et al*: Dissociation between clearances of small and middle molecules in incremental peritoneal dialysis. *Perit Dial Int* 21:462–466, 2001
32. De Smet R, Glorieux G, Hsu C, Vanholder R: P-cresol and uric Acid: Two old uremic toxins revisited. *Kidney Int* 52(Suppl 62):S8–S11, 1997
33. Lesaffer G, De Smet R, Lameire N, *et al*: Intradialytic removal of protein-bound uraemic toxins: role of solute characteristics and of dialyser membrane. *Nephrol Dial Transplant* 15:50–57, 2000
34. Maeda K, Shinzato T, Ota T, *et al*: Beta-2-microglobulin generation rate and clearance rate in maintenance hemodialysis patients. *Nephron* 56:118–125, 1990
35. Odell RA, Slowiaczek P, Moran JE, Schindhelm K: Beta$_2$-microglobulin kinetics in end-stage renal failure. *Kidney Int* 39:909–919, 1991
36. Xu XQ, Gruner N, Al-Bashir A, *et al*: Determination of extra renal clearance and generation rate of beta2-microglobulin in hemodialysis patients using a kinetic model. *Artif Organs* 47:623–627, 2001
37. Evenepoel P, Claus D, Geypens B, *et al*: Amount and fate of egg protein escaping assimilation in the small intestine of humans. *Am J Physiol* 277:G935–G943, 1999
38. Birkett A, Muir J, Phillips J, *et al*: Resistant starch lowers fecal concentrations of ammonia and phenols in humans. *Am J Clin Nutr* 63:766–772, 1996
39. Cummings JH, Hill MJ, Bone ES, *et al*: The effect of meat protein and dietary fiber on colonic function and metabolism. II. Bacterial metabolites in feces and urine. *Am J Clin Nutr* 32:2094–2101, 1979
40. Zehnder C, Gutzwiller JP, Renggli K: Hemodiafiltration—A new treatment option for hyperphosphatemia in hemodialysis patients. *Clin Nephrol* 52:152–159, 1999
41. Mucsi I, Hercz G, Uldall R, *et al*: Control of serum phosphate without any phosphate binders in patients treated with nocturnal hemodialysis. *Kidney Int* 53:1399–1404, 1998
42. Gallar P, Ortega O, Gutierrez M, *et al*: Influencing factors in the control of phosphorus in peritoneal dialysis. Therapeutic options. *Nefrologia* 20:355–361, 2000
43. Gutzwiller JP, Schneditz D, Huber AR, *et al*: Estimating phosphate removal in haemodialysis: an additional tool to quantify dialysis dose. *Nephrol Dial Transplant* 17:1037–1044, 2002
44. Ho-Dac-Pannekeet MM, Koopmans JG, Struijk DG, Krediet RT: Restriction coefficients of low molecular weight solutes and macromolecules during peritoneal dialysis. *Adv Perit Dial* 13:72–76, 1997
45. De Smet R, David F, Sandra P, *et al*: A sensitive HPLC method for the quantification of free and total p-cresol in patients with chronic renal failure. *Clin Chim Acta* 278:1–21, 1998
46. Hida M, Aiba Y, Sawamura S, *et al*: Inhibition of the accumulation of uremic toxins in the blood and their precursors in the feces after oral administration of Lebenin®, a lactic acid bacteria preparation, to uremic patients undergoing hemodialysis. *Nephron* 74:349–355, 1996
