# Supplementary material for: Peritoneal dialysis modality transition and impact on phosphate and potassium serum levels
Source: PLoS One. 2021 Oct 15;16(10):e0257140. doi: 10.1371/journal.pone.0257140 (PMC8519456; doi:10.1371/journal.pone.0257140)
Supplement: Attachment — Submitted filename: Response to reviewers-plos.docx [file pone.0257140.s004.docx]

Response to reviewers

PONE-D-21-02597
**Peritoneal dialysis modality transition and impact on phosphate and potassium serum levels.**

*Funding Statement.*

The funders had no role in study design, data collection and analysis, decision to publish, or preparation of this manuscript.

“The funder provided support in the form of salaries for AR, but did not have any additional role in the study design, data collection and analysis, decision to publish, or preparation of the manuscript. The specific roles of this author are articulated in the ‘author contributions’ section.”

The commercial affiliation of JWL, Fresenius Medical Care, did not play any role in this study and is actually a competing company of the original funder (Baxter Healthcare), The author was invited to contribute to the manuscript given his expertise in this field (epidemiologic/cohort studies) and because good previous collaborations.

5. Please amend the manuscript submission data (via Edit Submission) to include author Guilherme Yokoyama and Silvia Carreira Ribeiro

Sorry for the error, corrected in this submission

*2. Please provide clinical trial registration information for the associated BRAZPD II clinical trial.*

The BRAZPD study is a cohort study designed between July 2004 and 2011, prior to the contemporary era when registration in website as the clinicalstrials.gov is recommended for observational studies. The study was locally registered under the number MS n°25000.187284/2004-01.

**Reviewer #1:** 
**The authors mention that all data is available either in the manuscript or the supplemental materials. There appear to be no supplemental materials, and the only data presented in the manuscript is means and standard deviations. This is not making all data publicly available. The authors should repose their raw data, or indicate why doing so is not feasible (includes protected health information).**

Sorry for not making it clear. The BRAZPD study was designed between July 2004 and 2011, prior to the contemporary era when data sharing was strongly encouraged. Consequently, data sharing was not explicitly stated in the patient information and consent form, such that there are ethical and legal barriers to publicly sharing the data. The data contain potentially identifying patient and clinical information. Data requests should be addressed to the study coordinator at the Pontificia Universidade Catolica do Paraná.

**Not enough information is provided in the statistical analysis section. Chi-square tests, t-tests, and Wilcoxon methods are mentioned as being used. However, it is not clear where any of these tests were run, as there do not appear to be any Confidence intervals or p-values reported from any of those tests. To be reproducible, the reader should be easily able to identify what type of test was run for what comparison, and be able to assess whether those results were significant or not. That is completely missing from this manuscript**.

Sorry for not been clear in this section of the manuscript. Those tests were used to compare the study population with the original cohort and we didn´t report p values in Table 1 because this practice has been abandoned for some (DOI: 10.1016/j.joca.2018.07.017) (DOI: 10.4103/2155-8213.190481) and the STROBE statement doesn´t ask for p values for the descriptive data (Checklist - Item 14). To avoid both problems (the one raised by the reviewer and the issue associated with reporting p values for descriptive analysis we removed the mention to theses tests keeping only the tests used for the outcomes of interest).

**The authors mention in the results/discussion sections that sensitivity analysis was performed. However, there is no mention of this being done in the statistical analysis section.** **The statistical analysis section should include mention of how the sensitivity analysis was done.**

We included the proper information in the statistical analysis section.

**Also, more information is needed about the analysis of the cross-over data. Simply stating what statistical software package was used for the analysis is insufficient.** **What statistical method(s) was used to analyze the data, and how was that analysis performed? That is what needs describing, in addition to including the package used to perform the analysis. Overall, more detail is needed.**

The test uses analysis of variance. We included the link to all methods used for the command pkcross in our references.

**Figures 3 and 5 need more explanation. What is the dashed black line? How was the fractional polynomial prediction performed? How were the 95% CI's obtained from that fit? This information should be provided in the statistical analysis section.** **Additionally, are the gray dashed lines for the solid black line or the dashed black line? What are the boxplots depicting? Mention of them is not included in the figure description. For Figure 5B, there is no mention of portion B in the figure description.**

The dashed line represents the values that would be obtained in case of a perfect correlation between the pre and post values (45° angle). The fractional polynomial was estimated using the regression models described by Royston and Altman in 1994 (<https://doi.org/10.2307/2986270>). The boxplots depict the mean value, interquartile range and min. max. values of potassium during the time on CAPD and APD. Figure 5B now is mentioned in the legend.

Reviewer #2: This is an interesting and well-written paper.
My major comments:

1. In the Abstract you state " In this multicentric prospective cohort study. we selected all patients who remained on a PD modality for at least 6 months and switched PD modality." whereas in the Introduction section you write " Therefore, we designed a cross-over analysis of a nationally representative cohort to evaluate the impact of PD modality short-term changes on both serum potassium and phosphate levels. ". I am confused. BRAZPD was a multicentric prospective observational cohort study, but this paper is actually a cross-over analysis evaluating the impact of PD modality short-term changes on both serum potassium and phosphate levels. It would be nice to make this distinction clear for the readers.
 Thank you for your comments, we adjusted the manuscript to avoid the misinterpretation that the original version was causing.

2. The Abstract and Final conclusion is "The effect of a higher removal of middle size molecules as result of PD modalities in terms of clinical and patient-reported outcomes should be further explored ". I do not fully agree with the conclusion and refer to the 2003 KI paper (Removal of middle molecules and protein-bound solutes by peritoneal dialysis and relation with uremic symptoms; VOLUME 64, ISSUE 6, P2238-2243, DECEMBER 01, 2003) by Bert Bammens, Pieter Evenepoel, Kristin Verbeke, Yves Vanrenterghem (Attached a pdf of the article). I do not fully agree with the conclusion as Bammens' paper already suggested that protein-bound solutes were involved in the pathophysiology of uremic symptoms. During peritoneal dialysis p-cresol behaves like β2m, probably due to its protein binding. The total clearance of both molecules is significantly lower as compared to water-soluble solutes and mainly depends on residual renal function. Your study may confirm earlier findings by Bammens et al. Do you agree ? I consider their paper an important reference to yours.

Thank you for raising this topic and bring this new reference that we missed. We agree that Bammens manuscript complement our findings particularly in terms of the differentiation of water soluble and protein-binded molecules and as suggested we added this reference and adjusted our manuscript. In the other side, the study of Bammens et al also didn´t explored clinical outcomes or patient reported outcomes so we believe we could keep the message that both findings (ours and Bammens) should be tested for patient survival, quality of life and other outcomes.

3. How did you consider a prescription equivalence in dialysis effectiveness when a patient was converted from CAPD to APD or vice-versa ? For example, did all CAPD patients on 4 exchanges with only 1.5% Dianeal PD solutions converted to 10 liters 1.5% Dianeal PD solution exchanges during the night and 2 liters 1.5 % Dianeal during the day (long dwell) ? Probably not, right ? Any changes in prescription may have affected the clearances; especially total daily infused volume, total daily dialysis time (CAPD usually 24 hours, but in APD you might have a "dry day" or even an extra exchange). This could be a limitation of the study.

We agree and considered this as a key question. Knowing this equivalence would be of tremendous help making us able to establish a guide about how properly modify or adapt PD prescription when changing from one modality to another. Unfortunately many factors are involved, including patient (e.g., membrane profile) and prescription´s characteristics, and I don´t thing we will have this answer in the near future. This limitation was added to the manuscript and now read:

“in addition, we are aware that when a patient is convert from one PD modality to another it is very difficult to define equivalence because many confounding factors are presented, particularly patient´s membrane profile and the dwell time; finally…”

4. It would be important to describe the reasons for conversion from CAPD to APD and vice-versa ? For example, in the case of peritonitis, were the analysis made after complete remission of the peritonitis?

Peritonitis is indeed an important clinical event that may interfere with solute removal. We retrieved the number of episodes of peritonitis occurred within the timeframe of modality change in our study. Sixty five percent of the study population didn´t have any peritonitis episode reported. Of the remaining 35% this is the distribution between the episode and the time of modality exchange:

All dots above the continuous black line represents peritonitis episodes that occurred after modality change. All dots below the dashed black line are peritonitis episodes that occurred at least 3 months before modality change. So, in our opinion, for this study peritonitis episodes doesn´t seem to have been the driven factor for modality conversion. We added this message in our limitations: *”we explored a potential association between peritonitis episodes and the modality conversion (no association was found). However, we cannot completely rule out the possibility that for some small group of patients the conversion could have occurred during the remission phase.“*

5. Did you analyse the effect of the time-window (vintage) from the start of PD (CAPD or APD) to the day the patient was "enrolled" in this specific study ? I can imagine that some occur within a short time-window and others within a long time-window.

We didn´t but we checked after your comments. The vast majority changed modality within the first year of dialysis. Below the histograms showing time to transference for all patients and according to the subgroup.

Vintage overall population:

CAPD to APD

APD to CAPD

6. In the Introduction section you very nicely describe that "Although phosphate is only 96 Daltons, the phosphate molecule is
surrounded by an aqueous cover and it behaves as a middle size molecule ". However, in the Material and Methods section, you describe " Our objective was to analyze whether a change in the PD modality associates with the levels of two routine biochemical laboratories, namely potassium (a small size molecule) and phosphate (a middle size molecule)".
I suggest a change in this last statement from ".......and phosphate (a middle size molecule)" to......and phosphate (a small size molecule that behaves as a middle size molecule)".

Agreed, sorry for the mistake.

My minor comments:

1. In the affiliation of the authors in the first page, there must be some error for author Alfonso Ramos affiliation. It cannot be as it is now " Institution Alfonso, Mexico city, Mexico ". It is needed to be corrected.

Sorry for this error, his institution should now be shown correctly

2. BRAZPD has been fully supported/financed by Baxter Health Care and enrolling only Baxter PD patients in Brazil. Therefore, it is a surprise to see a Fresenius employee being a co-author. Has Fresenius Medical Care given any financial support to this specific study?

Dear Dr Jose Carolino, interesting and important question that deserves clarification. FMC didn´t contribute to this or any other manuscript of the BRAZPD. We´ve learnt that good science doesn’t have filiation and for this paper we followed the good examples of past experiences with the BRAZPD. Along the history of BRAZPD we always invite good people from outside Brazil that may contribute scientifically to the topic (Lambie, Qureshi, Finkelstein, McIntyre, Kirk and many others)

**Reviewer #3: The question of phosphate elimination remains important in peritoneal dialysis patients since strong dietary phosphate restriction may lead to malnutrition and phosphate binder intake may increase pill burden and the risk of side effects. Moreover, changes in serum potassium levels may increase the risk for life threatening complications. This multicentric prospective cohort study aimed to examine the influence of a switch from CAPD to APD and vice versa on serum phosphate and potassium levels and the results may contribute to better clinical understanding.**

Thank you for your comments and inputs, all of them were taken into account in this new version of the manuscript.

**Concerning the presentation of the key findings in Figure 3 A/B and 5 A/B the description of Figure 3 on page 13 and of Figure 5 on page 14 seems mismatching.**

Sorry for the mistake, the legends should now be presented correctly.

**It would be helpful if you could clarify this and/or add a more detailed description of Figure 3 and 5 as they are not self explaining especially with regard to the sample size.**

We improved the description of these figures to make it more clear to our readers. If we understood correctly, the differences in sample size is because the vast majority of patients started the therapy on CAPD and later on moved to APD. Transference from APD to CAPD are not normal in clinical practice because of some clinical characteristics (membrane profile) and patient´s preference (APD is normally associated to a better quality of life).
